# Pre- and post-bereavement experiences and support needs of family caregivers in hospital settings in Türkiye: a qualitative interview study

**DOI:** 10.1186/s12904-026-02125-w

**Published:** 2026-05-07

**Authors:** Çiğdem Fulya Dönmez, Mustafa Karaağaç, Ercan Bakır, Cara Bailey, Muzeyyen Seckin

**Affiliations:** 1 School of Medicine, Dentistry and Nursing, University of Glasgow, Glasgow, UK; 2 Faculty of Health Science, Mugla Sitki Kocman University, Fethiye, Türkiye; 3 School of Healthcare Services, Harran University, Sanliurfa, Türkiye; 4 Department of Nursing, Faculty of Health Sciences, Adıyaman University, Adıyaman, Türkiye; 5 Department of Nursing and Midwifery, School of Health Sciences, College of Medicine and Health, University of Birmingham, Birmingham, UK; 6 St Giles Hospice, Staffordshire, UK

**Keywords:** Family caregivers, Palliative care, Bereavement, Qualitative research

## Abstract

**Background:**

Timely and adequate bereavement support is associated with better mental health and psychosocial outcomes for bereaved individuals. There is limited research focusing on how the pre- and post-death process in the hospital setting influences family caregivers’ perceptions and support needs before and after bereavement. The aim of this study is to explore the pre-and post-bereavement experiences and support needs of family caregivers in hospital settings in Türkiye.

**Methods:**

A qualitative exploratory study using semi-structured in-depth interviews analysed thematically was conducted to provide a detailed understanding of the bereavement experience in the context of bereaved family caregivers’ in hospital settings. Purposive sampling was used to recruit bereaved family caregivers (*n* = 21) who had experienced caring for an adult patient with life-threatening conditions in hospital.

**Results:**

Twenty-one bereaved family caregivers participated, over half of the participants (62%) were female and, participant age ranged between 20 and 52 years, with the average age being 39.8 years. Four themes were generated: (1) Pre-bereavement process, (2) Communication during the dying stages, (3) Post-bereavement process and, (4) Bereavement support needs of family caregivers. Many caregivers reported that the lack of information about the disease process of their close person, unfinished business, and cultural barriers to the discussion of death made the bereavement process more difficult. Social support, culturally specific coping strategies, support from psychiatric consultation liaison nurses and empathetic communication were identified as facilitators of the bereavement process for family caregivers.

**Conclusions:**

We recommend a culturally sensitive family-centered approach, compassionate and empathetic communication, and the integration of psychiatric consultation liaison nurses into hospital bereavement care systems to provide dignified and culturally adapted bereavement support care in hospitals. The insights gained from the perspective of family caregivers may be utilised by mental health professionals and policy makers to improve hospital-based bereavement care. By exploring the perspectives of family carers in a middle-income country, our research contributes to a more inclusive understanding of bereavement support needs globally, particularly in culturally diverse contexts where research has been limited.

**Supplementary Information:**

The online version contains supplementary material available at 10.1186/s12904-026-02125-w.

## Introduction

In most developed countries, acute hospitals have an important role in the provision of palliative care. Evidence suggests that between 13% and 36% of hospitals meet the criteria for the palliative care needs of patients [1]. According to the results of an international study of 14 countries across 4 continents, deaths in hospital ranged from 25% to 85% [2]. In an 8-year observational study conducted in Türkiye, a developing country with an upper-middle income economy, reported that 77% of patients died in hospital [3]. In addition, due to the lack of palliative care units in Türkiye, people with life-threatening conditions are often admitted to hospital [4].

Family caregivers are an important resource in today’s healthcare systems and are estimated to provide approximately 60% of the total care needed in European Union countries [5]. However, there is an implicit reliance on caregivers in hospitals in low- and middle-income countries, who function as temporary health care providers during the patient’s hospital stay. This is significantly different from high-income countries, where professional care limits and shapes family caregivers’ roles in this setting [6]. For example, in Turkish culture, it is a common tradition for a family member to stay with the patient while they are in hospital. Without a family caregiver in the hospital, the patient is considered an ‘abandoned patient’. Therefore, in Türkiye, most family caregivers are extensively involved in patient care, staying at the patient’s bedside 24 h a day [7].

Delivering care for people nearing the end of their lives requires considerable effort and has a significant impact on the lives of caregivers [8]. Family involvement in hospital care may include washing, toileting, feeding, giving medication and wound care. In many cases, family caregivers also carry out other informal care roles, such as decision-making, financial support or communicating with health professionals about the patient’s situation and treatment [9]. In an integrative review [1], the hospital environment was reported to be noisy and busy, and criticised as an inappropriate place to die. Moreover, the sense of busyness within the hospital made patients and families feel ‘lost in the numbers’, leading them to feel worthless and uncared for [1]. Common challenges experienced by caregivers in different cultures include emotional distress and depression, reduced social contact, and financial difficulties [10]. Similarly, a recent qualitative systematic review found that challenges faced by family caregivers in hospital settings included caregiver burden, discouraging hospital environment, financial burden, ineffective communication with health professionals, lack of support and unpreparedness to provide care [9]. Additional issues occur when concerns about approaching death and a future without a loved one come to the fore. In a cross-sectional survey study, for example, 90% of caregivers reported that ‘completing tasks, resolving conflicts and saying goodbye’ were extremely important [11]. Moreover, the prevalence of unpreparedness before and after death is high, with 20% to 25% of caregivers feeling unprepared before and after bereavement [12]. This is of particular concern since unprepared caregivers are less accepting of death and may experience more depression, anxiety and prolonged grief disorder [12]. For this reason, family caregivers are defined as ‘hidden patients’ [13].

A qualitative study revealed that pre-bereavement communication and actions had an impact on the experience of bereavement - for instance, healthcare professionals supporting family caregivers before the death of their relative had a positive impact on the post-bereavement resolution process, whereas unhelpful words from others hindered recovery from bereavement [14]. Furthermore, a recent community-based cross-sectional study among bereaved family caregivers found a positive relationship between perceived quality of collaboration between healthcare professionals and family caregivers at the end of life and family caregivers’ emotional well-being after bereavement [15]. It is therefore clear that palliative care’s ability to enable continuity of care from before to after death can help prepare family caregivers for the approaching death of their close one and help them feel that they have not been abandoned or forgotten by the service after the close one has died [16].

While timely and adequate bereavement support and preparedness for death is associated with better bereavement outcomes, many family caregivers receive inappropriate support before and after the death of a close one [17, 18]. Palliative care provision tends to prioritise the support needs of patients for death rather than the pre-and post-bereavement support needs of caregivers [17]. It is crucial that palliative care providers need to pay more attention to the support needs of family caregivers, and to “caregrieving,” both while the patient is alive and after death [16].

Grief is a socially and culturally constructed process and each culture determines cultural norms for grief [19]. Existing knowledge on the bereavement experiences and support needs of family caregivers is largely derived from studies of Western societies and there is limited information on non-Western cultures [1, 20]. Moreover, a growing number of papers focused on the experiences of family caregivers caring for people with life-threatening conditions at home [21–23], however, there is little research focusing on how the pre- and post-death process in the hospital setting influences family caregivers’ perceptions and support needs before and after bereavement [24]. In addition, although family caregivers have a crucial role in the care of people with life-limiting illnesses, in many countries they receive little recognition and policy support for their efforts [4]. This study may help to reduce poor bereavement outcomes by contributing the provision of the family-centred bereavement care, as well as inform the development of hospital-based bereavement programmes for bereaved family caregivers. Furthermore, the findings of the study can increase healthcare professionals’ insights into the perspectives, preferences and needs of bereaved family members by taking into account the cultural differences of them.

Given this background, the current study aims to explore the pre-and post-bereavement experiences and support needs of family caregivers in hospital settings in Türkiye.

The objectives of this qualitative study are to investigate:

- How family caregivers managed the bereavement process before and after the death of their close one.
- Communication between family caregivers and healthcare professionals during the dying stages.
- Family caregivers’ perceptions of how they could have been supported before and after the death of their close one.

## Methods

### Theoretical background and literature positioning

Bereavement is a conceptualised and multidimensional process that extends beyond emotional grief to include social, cultural, and practical perspectives of the individual. The Dual Process Model (DPM) of Coping with Bereavement is a well-recognised theoretical framework for understanding how bereavement-related stressors, loss-oriented (directly related to grief and the emotional pain of losing a close one) and restoration-oriented (related to adjusting to changes in daily life, responsibilities, and identity following the loss), shape the grieving process [25, 26].

This oscillation between loss- and restoration-oriented coping is fluid, allowing individuals to engage with and withdraw from grief at different times, thereby facilitating adaptation [25, 26]. The DPM has been applied across a range of contexts to capture the complexity of bereavement experiences [26, 27]. However, research remains limited in relation to hospital-based experiences of family caregivers in Türkiye, where cultural norms and communication practices may differ from those in other countries due to variations in healthcare systems. These contextual factors may influence bereavement trajectories and shape how loss- and restoration-oriented coping processes unfold. Therefore, in this study, the DPM is employed as a useful lens through which to interpret the bereavement experiences of Turkish family caregivers in hospital settings following the death of a close one.

### Study design

A qualitative exploratory study [28] using semi-structured in-depth interviews was undertaken to provide a detailed understanding of the bereavement phenomenon in the context of bereaved family caregivers’ experiences in hospital settings [28–30]. This design is particularly relevant when information is needed directly from those experiencing the phenomenon under investigation [28, 29]. Using this design allowed to enhance the exploration of participants’ perceptions of complex and sensitive issues [24]. The consolidated criteria for reporting qualitative research (COREQ) was used to support rigour and transparency in the design [31].

### Population

The target population was bereaved family caregivers who have experienced caring for an adult patient with life-threatening conditions such as cancer, congestive heart failure, chronic obstructive pulmonary disease or dementia [32] in a hospital in Türkiye. In this study, family caregivers are defined as someone who is close to the patient; this individual(s) may be a spouse, relative, adult children or friend [33].

### Sample and recruitment

Purposive sampling was used to enrol bereaved family caregivers. Purposive sampling implies the intention to select carefully the specific types of respondents who can optimally enhance the better understanding of the issues under study [34]. In order to reach the participants, social media was utilised to find potential participants using flyers including aim of the study, contact information of one researcher and inclusion criteria. Once initial participants were recruited, the team decided whether to recruit additional participants via snowball sampling to ensure relevant diversity (in terms of age, gender and level of education) among participants [34]. The participants interested in participating were contacted by one of the researchers (MK) via e-mail or phone. After written and verbal consent was obtained, participants were invited to participate in semi-structured face-to-face interviews. None of the participants were known to the research team before the recruitment.

The inclusion criteria were as follows: (1) being an adult caregiver (over 18 years of age), (2) being directly involved in patient care before she/he died in a hospital setting, (3) family caregivers who have experienced the death of a close one over 18 years of age (i.e.: parent, adult children, sibling, aunt, uncle, grandparent, niece, nephew or friend) and 4) at least 6 months but not more than 24 months following the death. Exclusion criteria were as follows: (1) family caregiver who have dementia and (2) not willing to participate in the study.

### Data collection

Twenty-one (*n* = 21) in-depth, semi-structured face to face interviews were performed by three authors, the EB, ÇFD (a male and female assistant professor experienced in qualitative studies within palliative care) and MK (a male internal medicine research nurse with 7 years of experience working in hospital settings). The interviews were finalised when no further themes were identified to address the study aim [35]. This qualification criterion was based on previous studies focusing on data saturation for thematic analysis, given a relatively homogenous sample [17, 36, 37]. Interviews were carried out 6–24 months post bereavement between June and December 2024. The purpose of the timing of the interviews was to avoid interviewing immediately after the death of a close one and thus causing distress to the caregiver [29, 38, 39]. This was also the most preferred time period for the interview and parents reported that this time period enabled them to still remember very clearly what happened, how they felt and what they needed [39]. In accordance with the aims and objectives of the study, interview guide questions were designed based on the literature [15, 17, 18, 24]. To test the appropriateness of the questions, three pilot interviews were conducted and no significant modifications were needed (see Supplementary Material 1).

The interviews conducted in the participant’s home and were scheduled according to the availability of the participants. No other people were present during interviews. The interviews were audio-recorded and lasted between 35 and 72 min. The average duration of interviews was 45 min. The semi-structured face-to-face interviews gave participants the opportunity to detail and explain the specific issues [36]. The meetings started by questioning the participants about their experiences as a caregiver for their close one before and after he/she died (see interview guide in Supplementary Material 1). Initial responses were probed for more information (e.g. ‘Can you tell me more about this?’ and ‘Can you give me an example?’). Demographic data on the participants (Table 1) were collected through a questionnaire that was filled out by the participants before starting the interview. The interviews were performed by MK, ÇFD, and EB in Turkish and independently translated into English by two authors (ÇFD, MS) after the data analysis.

### Data analysis

Data was analysed thematically; a flexible method that enables the reporting of participants’ experiences, meanings and realities [40]. The six steps of Braun and Clarke’s reflexive thematic analysis was used by two researchers (ÇFD; MS); (1) familiarising with the dataset, (2) coding, (3) generating initial themes, (4) developing and reviewing themes, (5) refining, defining and naming themes and (6) writing up [40]. All data were independently read, and then coded using NVivo (Version 14) by the first author with qualitative research experience within palliative care (ÇFD) and a researcher with qualitative research experience within palliative care (MS). To discuss generated themes and resolve differences, regular research team meetings were scheduled, which resulted of the renaming of two main themes and two sub-themes. Disagreements in coding were resolved by CB (a professor with research experience in palliative care and qualitative methods).

### Reflexivity

The researchers have a professional background in nursing and formal training in qualitative research methodologies. The researchers’ positionality was explicitly acknowledged throughout the study to enhance transparency and reflexivity. No prior relationship existed between the researcher and participants, thereby minimising the potential for bias arising from pre-existing interactions or familiarity. The involvement of multiple researchers in coding, translation, and theme verification, alongside ongoing discussion within the research team, further contributed to the credibility, dependability, and confirmability of the study findings.

## Results

A total of 21 family caregivers met the inclusion criteria. Over half of the participants (%62) were female and, participant age ranged between 20 and 52 years, with the average age being 39.8 years. At participation, most families (76%) were 10–24 months into their bereavement. The relationship to the deceased varied, including grandson (*n* = 6), granddaughter (*n* = 5), daughter (*n* = 5), nephew (*n* = 3), and niece (*n* = 2) of the deceased. The participants’ sociodemographic characteristics can be found in Table 1.

**Table 1:** Demographic characteristics of bereaved individuals (*n* = 21)

Characteristics		
Gender	Female, *n*(%)	13 (62)
	Male, *n*(%)	8 (38)
Age	Mean (SD)	39.8 (6.7)
	Median (min-max)	42 (20–52)
Marital status	Married, *n*(%)	11 (52)
	Single, *n*(%)	10 (48)
Highest level of education	Undergraduate, *n*(%)	13 (62)
	High school, *n*(%)	6 (28)
	Secondary School, *n*(%)	1 (5)
	Postgraduate, *n*(%)	1 (5)
Relationship to the deceased	Grandson, *n*(%)	6 (28)
	Granddaughter, *n*(%)	5 (24)
	Daughter, *n*(%)	5 (24)
	Nephew, *n*(%)	3 (14)
	Niece, *n*(%)	2 (10)
Broad diagnostic category causing death	Congestive heart failure, *n*(%)	8 (38)
	Cancer, *n*(%)	8 (38)
	Chronic obstructive pulmonary disease, *n*(%)	3 (14)
	Dementia, *n*(%)	2 (10)
Age of the deceased	Mean (SD)	63.9 (9.3)
	Median (min-max)	58 (52–87)
Months since death to interview	Mean (SD)	14.7 (6.2)
	Median (min-max)	12 (6–24)

### Themes and subthemes

The research findings identified in depth the pre-and post-bereavement experiences and support needs of family caregivers in hospital settings. Four main themes and ten subthemes generated from the study analysis. The main themes were: *(1) Pre-bereavement process*,* (2) Communication during the dying stages*,* (3) Post-bereavement process *and,* (4) Bereavement support needs of family caregivers*. Each theme included between two and four subthemes illustrated by representative quotes from participants. Further data supporting these themes and sub-themes are provided in Table 2.

**Table 2:** Overview of qualitative themes

Major themes	Sub-themes	Illustrative quotes
Pre-bereavement process	• Family caregivers’ perceptions on healthcare delivery	“When I couldn’t go to the hospital to care her (my grandmother), my mind was on her. Because I knew that she was not well cared for in the hospital. For example, when she needed to go to the toilet, she had to hold her urine because there was no one to help her. When he called the nurses, they didn’t come. Or when she didn’t want to eat, no one was even trying to feed her. She was crying by telling us these things. She was constantly telling my aunt and father that you left me alone in the hospital, no one was coming to me. Maybe this is not an obligation, but the nurses still should help a little… I think, people who are near death deserve better care…” Participant 21 “If the healthcare delivery continues like this, cancer patients will die not from the disease but from the hospital process. The bad health system kills more than the disease. Taking a queue in the hospital, finding a bed for the patient, etc. procedures are very long and tiring.” Participant 17 “Nurses expect relatives of patients to help with their relatives’ needs, such as using the toilet and eating. I can not imagine what would happen if we were not at our relatives’ bedsides in hospital. I suppose nurses expect us to take on these kinds of tasks because they have such a heavy workload. ” Participant 20
	• Influence of culture on caregiver’s burden	“You know, in Turkish culture, we cannot leave our relatives in the hospital even if the doctors tell you that you can go home. Me and my mum stayed in the hospital for about a month, I took leave from my job. It is really exhausting to stay in the hospital without eating and sleeping properly.” Participant 21 “The last three months have been especially difficult. My mum became even more agitated… She was projecting her anger on me, and it was exhausting. When no one in the family took care of her properly, the burden fell on me…You know, women usually take on the burden of care. Washing, toileting and feeding were all left to me…” Participant 17 “…Everyone around me thought that, because I am a woman, it was my responsibility to care for my mother. Unfortunately, men do not feel as responsible in this regard as women do.” Participant 16
Communication during the dying stages	• “Death taboo” in cultural context	“…Although I knew that my father cancer had spread throughout his body, I didn’t want to think about death. Neither my family members, my dying father nor the nurses, doctors mentioned the word death even once. It is like a forbidden word (death) in our culture. So, the day my father died; I couldn’t believe it and didn’t speak to anyone for days…” Participant 21 “It is still very hard for me to believe that my mum is dead. My mum was sick before, she had diabetes and vertigo. Especially, when she had vertigo, I was with her whenever she needed care. Then, my mother got better. So, when my mother was diagnosed with cancer, I thought that this would pass, and my mother would get better again. Neither the nurses nor the doctors told us about death. So, it never even came to my mind that she would die.” Participant 16 “How can I possibly prepare for the death of my loved one when I am afraid even to say the word ‘death’? ” Participant 10
	• Lack of information	“Everything would have been easier for us if we had been given information about my grandmother’s disease process. Because we didn’t know what to do. Imagine you have a relative who is dying, you are trying to help him/her without even knowing what you are doing…” Participant 20 “Although the nurses provided good care for my grandfather during his time in intensive care, they did not provide us with sufficient information in response to our questions about his condition. ” Participant 8
Post-bereavement process: “I don’t know when this pain will ever go away”	• Bereavement reactions	“It’s been a year since my father died, but the pain hasn’t gone away. I still don’t sleep well; some nights I dream and wake up crying. I miss the smell of my father. I don’t know when this pain will ever go away.” Participant 21 “My grandmother’s death affected me a lot. I felt as if my childhood was slipping away from me, because as I said, he was in both the best and the worst memories of my childhood. As I said, I can say that it affected me a lot because I had a close relationship with my grandmother. I remember crying on the stairs of the hospital the day she died. It was also difficult for me to see my father and uncles devastated with sadness.” Participant 18 “After my grandmother died, I was left with a huge sense of emptiness. I miss her so much. Nothing can fill the hole she left behind.” Participant 12
	• Culture-Specific Coping Strategies	“After the funeral ceremony is over, there are condolence houses. In our culture, you sit in condolence houses and do not get up for 3 days, and you receive condolence of those who come and go from morning to evening. This process usually lasts 40 days…so think of it as 40 days of mourning. You don’t switch on the television, you don’t cut your hair or beard, you just talk about her (the deceased) all the time. After the 40th day, it is like a holiday…” Participant 19 “… I can say that ‘Mother Elif’ is a saint in our culture- a pir, someone who has a tomb. I went Mother Elif’s tomb on the last day before my grandmother died. Because, she was in great pain and cold sweat, I felt that she was dying (Sighing). So, I went to Mother Elif, I held her hand, I said please, and I wanted my grandmother to die without pain… In this way, my grandmother died peacefully. Before she died, I stroked my grandmother’s head, wiped her sweat, kissed her, and said goodbye, saying that Mother Elif would be your comrade. I am glad that there is Mother Elif to help us.” Participant 18
	• Social Support	“Having unity and solidarity in difficult times keeps people alive. When my mother died, it was good for all of us to come together with nearly 130 cousins. At my grandmother’s funeral there were even some cousins and relatives we had never met before. We took each other’s phones and tried to be with each other by establishing stronger ties.” Participant 19 “In this process, it is a very valuable thing to feel the presence of people close to you that you can talk to…This can be a friend, a mother, a sister or a wife.” Participant 10 “What gave me strength was the support of relatives and friends who never left me alone during those days.” Participant 17
	• Unfinished business	“I spent a lot of time with him before he (my uncle) died, but I never told him I loved him. We had things we planned to do together. Unfortunately, we didn’t do any of them, and I’m very sad that I lost him so early. ” Participant 2 “I would have loved to have her (my grandmother) with me when I got married. I would have loved to have fun together at my wedding, but it didn’t happen…” Participant 18 “…I regret not telling her often enough how much she meant to me.” Participant 16
Bereavement support needs of family caregivers	• Support from psychiatric consultation liaison nurses	“The number of consultation liaison nurses in Turkiye is very limited. I think this process can be overcome much more easily if support is given from consultation and liaison nurses before and after death. ” Participant 21 ‘’It would be easier to cope with this difficult process if we had nurses who were trained to provide psychological support to us and our loved ones. Participant 7
	• The need for a compassionate and empathic communication before and after death	“When we went to the emergency room, my father was already dead. My feet were bare, I even forgot to wear shoes in my panic. A nurse brought me slippers even though she didn’t know me. That behaviour of the nurse was very healing for me, it still stays in my mind.” Participant 7 “Health professionals should be able to empathise with us in this difficult process and approach us with compassion by understanding our psychology.” Participant 21 “The little things that nurses did, like saying kind words or gently touching me, made me feel much better during that difficult time.” Participant 17

### Theme 1: Pre-bereavement process

The theme of pre-bereavement process included the following sub-themes: (1) Family caregivers’ perceptions on healthcare delivery, and (2) Influence of culture on caregiver’s burden.

#### Family caregivers’ perceptions on healthcare delivery: “The bad health system kills more than the disease”

Family caregivers who participated in our study reported that the issues in the healthcare system made their bereavement process difficult. Some of the caregiver families emphasised that they thought that their relatives approaching the end of life could not receive sufficient healthcare in the hospital. For example, one participant emphasised that nurses neglect to meet the basic needs of her relative and this causes an emotional burden on both the patient and the family member as well as suggesting that these patients deserve more compassionate and dignified care:

*“When I couldn’t go to the hospital to care her (my grandmother)*,* my mind was on her. Because I knew that she was not well cared for in the hospital. For example*,* when she needed to go to the toilet*,* she had to hold her urine because there was no one to help her. When he called the nurses*,* they didn’t come. Or when she didn’t want to eat*,* no one was even trying to feed her. She was crying by telling us these things. She was constantly telling my aunt and father that you left me alone in the hospital*,* no one was coming to me. Maybe this is not an obligation*,* but the nurses still should help a little… I think*,* people who are near death deserve better care…”* (Participant 21).

Some participants expressed deep frustration with the delivery of health care, emphasising that inefficiencies and poor hospital management can be more damaging than the disease itself. One participant emphasised that long waiting times, difficulties in accessing hospital beds and general bureaucratic delays were particularly distressing for patients with life-threatening conditions such as cancer:

“If the healthcare delivery continues like this, cancer patients will die not from the disease but from the hospital process. The bad health system kills more than the disease. Taking a queue in the hospital, finding a bed for the patient, etc. procedures are very long and tiring” (Participant 17).

#### Influence of culture on caregiver’s burden: “It’s exhausting”

Culture significantly influences the role of family members in care. Some participants mentioned that they try not to leave their relatives alone by staying in the hospital and emphasised the strong family ties in Turkish culture. However, this can cause emotional, physical and economic burden of care, including sleep disturbance, not being able to eat properly and not being able to go to work as Participant 21 highlights:

*“…in Turkish culture*,* we cannot leave our relatives in the hospital even if the doctors tell you that you can go home. Me and my mum stayed in the hospital for about a month*,* I took leave from my job. It is really exhausting to stay in the hospital without eating and sleeping properly”* (Participant 21).

Some participants also emphasised that the burden of care is mostly on women which makes this stressful process more difficult. One of the caregiver families said:

*“The last three months in hospital have been especially difficult. My mum became even more agitated… She was projecting her anger on me*,* and it was exhausting. When no one in the family took care of her properly*,* the burden fell on me…You know*,* women usually take on the burden of care…”* (Participant 17).

### Theme 2: Communication during the dying stages: “Forbidden word”

Family caregivers described their experiences of communication during the dying stages in two sub-themes: (1) “Death taboo” in cultural context, and (2) Lack of information.

#### “Death taboo” in cultural context

Most family members said that they were not prepared for the death of their close one. The fact that death is taboo in Turkish culture causes even healthcare professionals not to talk about it. This leads to healthcare workers not being able to prepare families for the death of their relatives. In the example below, the words of a participant who still could not believe in the death of her relative clearly express this situation:

*“…Although I knew that my father’s cancer had spread throughout his body*,* I didn’t want to think about death. Neither my family members*,* my dying father nor the nurses*,* doctors mentioned the word death even once. It was like a forbidden word. So*,* the day my father died; I couldn’t believe it and didn’t speak to anyone for days…”* (Participant 16).

#### Lack of information

Some participants mentioned that they were not given adequate information about the disease process of their relatives approaching the end of life. Participant 20 highlights the importance of clear communication in helping family members cope and provide better support to their close ones as others did:

*“Everything would have been easier for us if we had been given information about my grandmother’s disease process. Because we didn’t know what to do. Imagine you have a relative who is dying*,* you are trying to help him/her without even knowing what you are doing…”* (Participant 20).

### Theme 3: Post-bereavement process: “I don’t know when this pain will ever go away”

The theme of post-bereavement process included the following sub-themes: (1) Bereavement reactions, (2) Culture-Specific Coping Strategies, (3) Social Support, and (4) Unfinished business.

#### Bereavement reactions

The most typical grief reactions experienced by participants after the death of a close one were emotional pain and longing. Some of the bereaved participants reported that they were still experiencing sleep problems and intense feelings of grief more than a year after the death of their close one. One bereaved caregiver shared her emotional pain in the following words:

*“It’s been a year since my father died*,* but the pain hasn’t gone away. I still don’t sleep well; some nights I dream and wake up crying. I miss the smell of my father. I don’t know when this pain will ever go away.”* (Participant 21).

Similarly, another participant emphasised that the death of a close one, especially one closely linked to childhood memories, causes deep emotional pain:

*“My grandmother’s death affected me a lot. I felt as if my childhood was slipping away from me*,* because as I said*,* he was in both the best and the worst memories of my childhood…As I said*,* I can say that it affected me a lot because I had a close relationship with my grandmother. I remember crying on the stairs of the hospital the day she died…”* (Participant 18).

#### Culture-specific coping strategies

The experiences of the participants in this study reveal the importance of a comprehensive and culturally sensitive perspective in understanding the unique needs of caregivers. Participants mentioned some culture-specific rituals to cope with the grieving process. For example:

*“After the funeral ceremony is over*,* there are condolence houses. In our culture*,* you sit in condolence houses…You receive condolence of those who come and go from morning to evening. This process usually lasts 40 days…so think of it as 40 days of mourning. You don’t switch on the television*,* you don’t cut your hair or beard*,* you just talk about her (the deceased) all the time. After the 40th day*,* it is like a holiday…”* (Participant 19).

The findings of this study show that some culture-specific beliefs may have an effect on the bereavement process. One of the participants emphasised the role of a respected figure such as ‘Mother Elif’, who is regarded as a saint (pir) according to her cultural beliefs, in supporting the bereavement process:

*“… I can say that ‘Mother Elif’ is a saint in our culture- a pir*,* someone who has a tomb. I went Mother Elif’s tomb on the last day before my grandmother died. Because*,* she was in great pain and cold sweat*,* I felt that she was dying (Sighing). So*,* I went to Mother Elif*,* I held her hand*,* I said please*,* and I wanted my grandmother to die without pain… In this way*,* my grandmother died peacefully. Before she died*,* I stroked my grandmother’s head*,* wiped her sweat*,* kissed her*,* and said goodbye*,* saying that Mother Elif would be your comrade. I am glad that there is Mother Elif to help us.”* (Participant 18).

#### Social support

Social support during bereavement process facilitates coping with grief and positively affects well-being. Most participants in this study emphasised the vital role of unity and togetherness in coping with the death of a close one, highlighting how social support can provide emotional strength in difficult times. One participant emphasised how family unity not only provided comfort in times of grief, but also strengthened relationships for the future:

*“Having unity and solidarity in difficult times keeps people alive. When my mother died*,* it was good for all of us to come together with nearly 130 cousins. At my grandmother’s funeral there were even some cousins and relatives we had never met before. We took each other’s phones and tried to be with each other by establishing stronger ties.”* (Participant 19).

#### Unfinished business

Bereaved family caregivers described specific concerns about unfinished business in the context of important things left unsaid or unresolved. Some expressed the sadness of not being able to tell their loved ones that they loved them before they died, others the pain of things they wanted to do together but couldn’t do. For example:

*“I spent a lot of time with him before he (my uncle) died*,* but I never told him I loved him. We had things we planned to do together. Unfortunately*,* we didn’t do any of them*,* and I’m very sad that I lost him so early.”* (Participant 2).

Another bereaved individual stated: *“I would have loved to have her (my grandmother) with me when I got married. I would have loved to have fun together at my wedding*,* but it didn’t happen…”* (Participant 18).

### Theme 4: Bereavement support needs of family caregivers

Family caregivers described their bereavement support needs in two sub-themes: (1) Support from psychiatric consultation liaison nurses, and (2) The need for a compassionate and empathic communication before and after death.

#### Support from psychiatric consultation liaison nurses

Some family caregivers have highlighted that having a sufficient number of consultation-liaison nurses in hospitals is critical for effective bereavement support. According to them consultation liaison nurses can help them for timely and supportive bereavement care, it helps to decrease distress and improve emotional healing. One family caregiver said: *“The number of consultation liaison nurses in Turkiye is very limited. I think this process can be overcome much more easily if support is given from consultation and liaison nurses before and after death. ”* (Participant 21).

#### The need for a compassionate and empathic communication before and after death

Most of the participants emphasised that compassionate and empathetic communication provided by health professionals before and after death was very important for them to overcome this difficult process. One family caregiver stated the importance of empathy from care professionsal:

*“Health professionals should be able to empathise with us in this difficult process and approach us with compassion by understanding our psychology.”* (Participant 21).

Compassionate and empathic communication can also create a safe space for them to go through the grieving process with dignity and support. For example Participant 7 reflected on the kindness and compassion shown from the emergency nurse helped her:

*“When we went to the emergency room*,* my father was already dead. My feet were bare*,* I even forgot to wear shoes in my panic. A nurse brought me slippers even though she didn’t know me. That behaviour of the nurse was very healing for me*,* it still stays in my mind.”* (Participant 7).

Our findings revealed that many caregivers faced inadequate communication, limited psychological support, and cultural barriers when discussing death.

## Discussion

To our knowledge, this is the first Turkish study to the pre-and post-bereavement experiences and support needs of family caregivers in hospital settings. The objectives were to investigate: (i) how family caregivers managed the bereavement process before and after the death of their close one., (ii) communication between family caregivers and healthcare professionals during the dying stages, and (iii) family caregivers’ perceptions of how they could have been supported before and after the death of their close one.

Family caregivers who participated in the present study, reported that the issues in the healthcare system made their bereavement process difficult. Some of the family caregivers expressed deep frustration with the delivery of health care, emphasising that inefficiencies and poor hospital management can be more damaging than the disease itself. In our study, in line with other qualitative studies [41, 42], it was found that the barriers related to the healthcare system made grief and bereavement experience difficult for family caregivers who have experienced caring for an adult patient with life-threatening conditions. A qualitative study conducted in Canada [41] found that most bereaved family caregivers were frustrated when their expectations of health professionals and the health system were not met, both during caregiving and long after the patient’s death. Family caregivers emphasised the negative impression that the lack of attention to personalised care, whether from an individual health professional, the health system at large or the related government agencies, had on them [41]. It is thought that the fast pace of the hospital environment has an important effect on these negative experiences of bereaved family caregivers [43]. Organisational change to reflect an institutional commitment to patient- and family-centred care is needed to address these system-wide barriers [44].

Family involvement in the care of hospitalised patients is generally considered as critical, with families advocating for their close ones and seeking to ensure that care is consistent with the patient’s preferences and values [45]. In Turkish culture, it is traditional for a family member to stay with the patient while they’re hospitalised and the patient who is not accompanied by a family member in the hospital is considered an “abandoned patient“ [7]. In the present study, some participants mentioned that they try not to leave their relatives alone by staying in the hospital and emphasised the strong family ties in Turkish culture. This study’s findings also showed that caring to the patient in hospital for a long time can cause emotional, physical and economic burden of care, including sleep disturbance, not being able to eat properly and not being able to go to work. In line with this, previous research [46, 47] has shown that being a family member of a person approaching the end of life can have a multidimensional impact: psychological, with increased stress and anxiety; physical, due to various practical care activities; financial, with having to leave work to be a caregiver; and social, with limitations on social life. Support for family caregivers is therefore an crucial part of palliative care [46].

In our study, most of the family members emphasised that they were not prepared for the death of their close ones and that it was very difficult for them to talk about death. They also reported that the word “death” was like a forbidden word, similar to the results of the studies in the Chinese cultural context [48, 49]. The fact that death is taboo in Turkish culture [50] causes even healthcare professionals not to talk about it. This cultural background can cause difficulties in health care settings, especially in end-of-life care, where effective communication about death is critical [49]. Hence, previous research has highlighted the need to support healthcare professionals in delivering quality palliative, end-of-life and bereavement care, through culturally sensitive interventions and improved communication strategies [48, 49].

Bereavement rituals, which provide psychological, social and cultural support, are crucial in the grieving process [51, 52]. They help to ease the adjustment process, provide psychosocial benefits and help to maintain links with the deceased [51, 53]. In this study, the experiences of the participants reveal the importance of a comprehensive and culturally sensitive perspective in understanding the unique needs of caregivers. Participants mentioned some culture-specific rituals to cope with the grieving process, the importance of rituals for bereavement process has been identified in other studies [51, 53].

Lastly, a lack of access to appropriate support, including compassionate and empathetic communication and support from mental healthcare professionals, was also reported in this and other studies [18, 54, 55]. The findings of this study reveal that cultural barriers, inadequate access to psychiatric consultation liaison nurses and inadequate support before and after bereavement negatively affect bereaved families socially, physically and psychologically [41]. These findings suggest unmet need in supportive bereavement care provision and follow up support [18]. Early palliative care, including a focus on the family caregiver and, where appropriate, referral to another healthcare professionals (e.g. psychiatric consultation liaison nurses) for counselling, appears to be a promising way to decrease family caregiver’s distress and meet their personal needs [56].

The DPM framework [25, 26] was particularly relevant in the Turkish cultural context, where family plays a central role in caring for individuals with serious illness and at the end of life. Discussions about dying and death may be constrained by cultural norms, while mourning practices are strongly influenced by dual religious and communal traditions [26, 57]. Based on the findings from our participants, this study captures not only the personal and emotional aspects of bereavement as loss- and restoration-oriented stressors and reactions, but also the sociocultural and systemic factors that shape coping strategies and the needs of caregivers. However, at the intersection of these dimensions, key themes emerged, including bereavement responses, unfinished business, culturally specific coping strategies, and the persistence of the “death taboo” within cultural contexts. These intersecting themes underscore the complexity of caregivers’ experiences, as they navigate the emotional burden of loss alongside practical and culturally mediated coping strategies. Furthermore, the hospital environment and caregiving practices, alongside these factors, mediate the oscillation between loss- and restoration-oriented coping. This positions the study to highlight the importance of integrating theoretical models of bereavement with practice-oriented recommendations, particularly to enhance hospital- and community-based bereavement support in contexts where family caregivers provide care for a close one with a serious illness over extended periods of time. A recent meta-analysis of randomised controlled trials indicated that bereavement support delivered by nurses and other healthcare professionals in palliative care settings is effective in reducing grief, depression, and anxiety following a loss [58].

Future research should examine the facilitators and barriers to communication between family caregivers and healthcare professionals during the dying phase and in bereavement support across community and hospital settings. Further investigation is also needed into how caregiving activities, duration, burden (physical, psychological, and financial), and preparedness for death influence bereavement outcomes and mitigate restoration stress for family caregivers across cultures. While culturally adapted bereavement support is recognised in the literature, greater clarity is required regarding its core domains and effective delivery. Advancing this evidence will support the development of robust, culturally sensitive bereavement care across palliative care services and hospices in different countries.

### What this study adds

Although timely and adequate bereavement support and preparedness for death is associated with better bereavement outcomes, many family caregivers receive inappropriate support before and after the death of a close one [17, 18]. Moreover, a growing number of papers focused on the experiences of family caregivers caring for people with life-threatening conditions at home [21], however, there is little research focusing on how the pre- and post-death process in the hospital setting influences family caregivers’ perceptions and support needs before and after bereavement [24]. Besides, the previous studies conducted in high-income countries [1, 20, 59, 60] and there is limited information on the bereavement support needs of family caregiver in a middle-income country. The findings contribute to the global understanding of bereavement from family caregiver perspective in culturally diverse populations. In addition, this qualitative study offers invaluable insights into the how family caregivers managed the bereavement process before and after the death of their close one. Our findings also shed light on supportive strategies including culturally sensitive, family-centered approaches from family caregivers’ perspective to help them before and after the death of their close one. Based on the principles of clinical bereavement support guidelines for caregivers in palliative care in Australia, Singapore, the USA, New Zealand, Canada, South Korea, and Ireland, support throughout the bereavement process, including the pre- and post-death periods, should include: “organising support for the family caregiver; assessing needs and establishing a care plan; ensuring information and support for the family caregiver; preparing for death; providing support at the time of death; and offering bereavement support post-death” [61]. These elements should be adopted to ensure timely referrals according to individual needs. This is possible through a focus on individualised support and care, with the inclusion of cultural perspectives and effective communication, as highlighted in our present qualitative study. The need for bereavement support has been emphasised, including the role of psychiatric consultation-liaison nurses, the importance of compassionate and empathetic communication both before and after death, and the necessity of holistic support that addresses both dimensions of the bereavement process in caregiving. As pre-bereavement collaboration between family caregivers and healthcare professionals positively improves the post-bereavement emotional well-being of family caregivers, the involvement of nurses and other healthcare professionals [15], alongside effective communication, is important not only in Türkiye but also in other countries.

### Strength and limitations

This is the first descriptive qualitative Turkish study to explore the pre-and post-bereavement experiences and support needs of family caregivers in hospital settings. The analysis process was conducted with analytical transparency, as indicated by the authors’ methodology for analysing and interpreting the data. To ensure confirmability, analysis was conducted by multiple authors. Furthermore, in-depth interviews were conducted with family caregivers following the death of their family member. This enabled the participants to reflect on their experiences and the support they required during the palliative phase. Presumably, our results are transferable to similar settings: middle-income countries where patients with palliative care needs are still treated in hospitals. However, our study also has several limitations. This study was conducted exclusively in Türkiye; consequently, it is only possible to present the spectrum of family carers’ experiences with the translation of interview data from Turkish to English. It should be noted that there is a risk that some meanings may be misinterpreted and misrepresented during the translation process. Additionally, in Turkish culture, end-of-life care is strongly shaped by family dynamics. Providing care is widely regarded as a family duty, with particular emphasis on children and, to a lesser extent, grandchildren [62]. This expectation is rooted in cultural norms that prioritise collective family responsibility over individual or spousal caregiving. Evidence shows that family members, especially adult children, play a central role in caregiving, decision-making, and communication at the end of life, often taking precedence over partners. As a result, the primary caregiver is frequently a child or another close relative rather than a spouse. This highlights the importance of recognising cultural values ​​and family structures in planning and implementing end-of-life care and bereavement support. Furthermore, as participants were recruited via social media, only those willing and able to respond were included. This may have excluded older carers who are less active in digital environments or less inclined to share their experiences publicly, thereby limiting the diversity of perspectives captured. The use of snowball sampling may also have constrained diversity, as individuals are more likely to recommend others within their own social networks, which can result in more homogeneous perspectives.

## Conclusion and implications for policy and practice

**Fig. 1:**
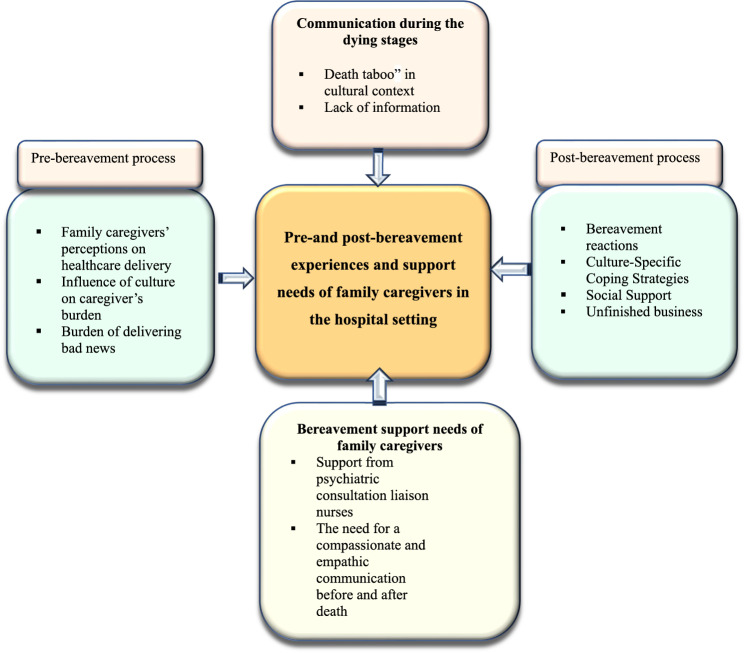
A proposed framework for pre-and post-bereavement experiences and support needs of family caregivers in the hospital setting

This study highlights the critical role of hospital-based pre- and post-bereavement support in shaping family caregivers’ experiences and bereavement outcomes. Our findings revealed that many caregivers encounter inadequate communication, limited psychological support, and cultural barriers in discussing death. The study also reveals the significant emotional burden faced by caregivers and the necessity for structured, compassionate interventions within hospital settings. Contrary to the findings of previous studies conducted in high-income countries, this study examines the experiences and needs of bereaved family caregivers in a middle-income country. The findings and proposed framework (Fig. 1) can contribute to the global understanding of bereavement from family caregivers’ perspective in culturally diverse populations. The insights gained from the perspective of family caregivers may be utilised by mental health professionals and policy makers to improve hospital-based bereavement care.

Based on the study findings, we make three recommendations: (i) a culturally sensitive family-centered approach including compassionate and emphatic communication to provide dignified and culturally adapted bereavement support care in hospitals; (ii) a structured bereavement support programme that includes hospitals, pre-bereavement counselling and post-bereavement follow-up to provide ongoing care for family caregivers; and (iii) the integration of psychiatric consultation liaison nurses within hospital palliative care teams has been proposed as a means of providing caregivers with timely mental health support, facilitating the management of pre- and post-bereavement challenges. Further studies are critically needed focusing on pre-and post-bereavement experiences and culture-specific support needs of family caregivers to provide tools/guidelines for all health care settings, from tertiary referral centres in high-income countries to resource-limited settings in low-income and middle-income countries.

## Supplementary Information

Supplementary Material 1.

## Data Availability

The datasets supporting the conclusions of this study are available from the corresponding author upon request.
