# Supplementary material for: Pre- and post-bereavement experiences and support needs of family caregivers in hospital settings in Türkiye: a qualitative interview study
Source: BMC Palliat Care. 2026 May 7;25:186. doi: 10.1186/s12904-026-02125-w (PMC13321540; doi:10.1186/s12904-026-02125-w)
Supplement: Supplementary file 1 — Supplementary Material 1. [file 12904_2026_2125_MOESM1_ESM.docx]

**Supplementary material**

**Supplementary material 1: Interview guide questions**

1. **Introduction**

• Thank you for agreeing to take part of our study.

• Give background & purpose to study: we want to explore your bereavement experiences and support needs before and after the death of your close relative in hospital.

This study can contribute to the development of hospital-based bereavement programmes for bereaved family caregivers in future and increase healthcare professionals' insights into the perspectives, preferences and needs of bereaved family members.

• Acknowledge that the topic is upsetting / sensitive. If they want to have a break at any time (and stop the recording) that is absolutely fine.

• Explain the consent procedure, right to withdraw, confidentiality and audio recording of the discussion. Break at any time if required. Interview discussion to last 30–90 min. Check that they have understood the information sheet, confidentiality information and check understanding.

• Explain how the discussion that is going to take place will be used in the research.

• Findings will be published in academic journals

• Complete consent forms

1. **I would really like to start by hearing about your experience before the death of your close one . Is that okay?**

- How was your experience as a caregiver for your close relative?
- How would the death of your close relative affect your life and psychology?
- If you had difficulties before and after the death of your loved one, what helped you cope with them?
- What was your experience in communicating with healthcare professionals during the dying stages of your close relative?
- What are your suggestions for improving bereavement support in hospital settings?

1. **Closing (Any other comments, suggestions or questions)**

- I would like to ask you for your final thoughts reflections as we come to the end of our discussion. I am grateful that you have shared your experiences. Your views are very valuable to us, and we hope that you have not found it too distressing to share your experience.
